# Pharmaceutical Aspects of Nanocarriers for Smart Anticancer Therapy

**DOI:** 10.3390/pharmaceutics13111875

**Published:** 2021-11-05

**Authors:** Seung Rim Hwang, Kushal Chakraborty, Jeong Man An, Jagannath Mondal, Hong Yeol Yoon, Yong-kyu Lee

**Affiliations:** 1 College of Pharmacy, Chosun University, 309 Pilmun-daero, Dong-gu, Gwangju 61452, Korea; srhwang@chosun.ac.kr; 2 Department of IT and Energy Convergence (BK21 FOUR), Korea National University of Transportation, Chungju 27469, Korea; Kushalchem1994@gmail.com; 3 Department of Bioengineering, College of Engineering, Hanyang University, Seoul 04763, Korea; jumpyoho@hanyang.ac.kr; 4 Department of Green Bio Engineering, Graduate School, Korea National University of Transportation, Chungju 27469, Korea; jagannathrkm@gmail.com; 5 4D Convergence Technology Institute, Korea National University of Transportation, Jeungpyeong 27909, Korea; 6 Center for Theragnosis, Biomedical Research Institute, Korea Institute of Science and Technology (KIST), Seoul 02792, Korea; seerou@kist.re.kr; 7 Department of Chemical and Biological Engineering, Korea National University of Transportation, Chungju 27469, Korea

**Keywords:** pharmaceutical, nanocarrier, cancer, smart, polymer, lipid, virus, inorganic, hybrid, electrospinning

## Abstract

Drug delivery to tumor sites using nanotechnology has been demonstrated to overcome the drawbacks of conventional anticancer drugs. Altering the surface shape and geometry of nanocomposites alters their chemical properties, which can confer multiple attributes to nanocarriers for the treatment of cancer and their use as imaging agents for cancer diagnosis. However, heterogeneity and blood flow in human cancer limit the distribution of nanoparticles at the site of tumor tisues. For targeted delivery and controlled release of drug molecules in harsh tumor microenvironments, smart nanocarriers combined with various stimuli-responsive materials have been developed. In this review, we describe nanomaterials for smart anticancer therapy as well as their pharmaceutical aspects including pharmaceutical process, formulation, controlled drug release, drug targetability, and pharmacokinetic or pharmacodynamic profiles of smart nanocarriers. Inorganic or organic-inorganic hybrid nanoplatforms and the electrospinning process have also been briefly described here.

## 1. Introduction

Nanoparticles with sizes ranging from tens to hundreds of nanometers in at least one of their dimensions demonstrate a large surface-area-to-volume ratio and quantum effects. Their large surface area enables them to have chemical reactivity for the attachment of multifunctional moieties [1]. Nanomaterials vary in dimension (from zero to three dimensions) and shape (from spherical to irregular shape), which affect their electrical, optical, or magnetic behaviors [2]. Various materials, including synthetic or natural polymers, lipids, viruses, carbon, and metals, have been investigated for their nanoparticle composition [3,4]. As the technology for controlling the size and biocompatibility of nanomaterials has advanced over recent years, nanomaterials have been extensively applied in various fields, including biomedical sciences [5].

**Figure 1:**
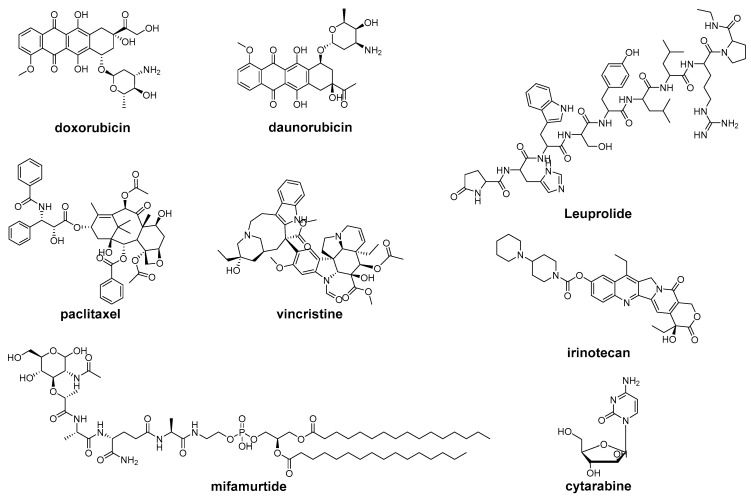
Molecular structures of doxorubicin, daunorubicin, leuprolide, paclitaxel, vincristine, mifamurtide, irinotecan, and cytarabine.

Nanocarriers for biomedical applications are typically larger than single molecules, such as water or glucose, and smaller than bacteria or cells. They can protect enclosed small-molecule drugs or imaging agents with relatively high loading efficiency. The concept involving the accumulation of nanoparticles around cancerous tissue with leaky vasculature due to enhanced permeability and retention (EPR) effect has been prevalent since the 1980s [6]. Nanoformulations based on polymers or lipids have been reported to enhance water solubility, permeability, and retention of drugs at the disease site [7]. Clinical applications of nanomedicines loaded with anticancer drugs such as doxorubicin (DOX), daunorubicin, leuprolide, paclitaxel (PTX), vincristine, mifamurtide, irinotecan, and cytarabine (Figure 1) into liposomes or albumin-coated nanoparticles have already been approved by the Food and Drug Administration (FDA) or European Medicine Agency (EMA) [8,9,10,11,12,13,14,15,16,17,18,19] (the list in Table 1 is not exhaustive).

However, the distribution of nanomedicines at the site of tumor tissues in humans with passive targeting using the EPR effect is limited. Penetration or accumulation of drugs in bulk tumors varies in humans due to interstitial fluid pressure [20]. In order to improve treatment outcomes and reduce systemic side effects, the surface of nanomaterials can be modified or functionalized with ligands for targeting cancer cells or for tailored therapeutic approaches [21]. Recently, anticancer nanotherapeutics using photosensitizers or those with a combined function of diagnosis and treatment have been developed. An inorganic nanomaterial and an anticancer agent can be hybridized, or a fluorescent material and an anticancer agent can be simultaneously loaded into one nanoformulation. Recently, inorganic nanoparticles have been also combined with radiotherapy [8].

When injected into the blood circulation of the body, nanoparticles smaller than 5.5 nm in their hydrodynamic diameter are primarily eliminated via renal clearance, but particles larger than 7 nm in their diameter cannot be easily eliminated by urinary excretion [22]. The size limit of the pore and negative charge around the glomerular capillary basement membrane prevent the filtration of large plasma proteins [23]. Without any special coating, liposomes with 100–200 nm in their diameter tend to become trapped in the liver or spleen [24]. As nanoparticles can bind to cellular receptors or are endocytosed by the cells, the release of anticancer agents from nanocarriers needs to be ideally controlled for smart anticancer therapy.

In this review, we categorize smart nanocarriers for anticancer therapy as polymer-based nanocarriers, lipid-based nanocarriers, inorganic nanocarriers, and hybrid nanocarriers. The electrospinning technique for the production of nanofibers in bulk is also introduced. Finally, we discuss the future directions of smart nanocarriers for effective cancer treatment.

## 2. Why Is a “Smart” Nanocarrier Needed for the Treatment of Cancer?

Owing to cell proliferation and angiogenesis, the blood vessels inside the tumors are abnormally shaped, and the endothelial cells have large fenestrations that are not aligned or organized [25]. Due to slow venous return and impaired lymphatic drainage, nanomedicines accumulated around cancerous tissue may remain within the tumor tissue [26]. However, intra-tumor heterogeneities in human patients and the extracellular matrix (ECM) surrounding the solid tumor tissue restrict the distribution of the nanomedicine attributed to the EPR effect through the leaky tumor vasculature [27,28]. The progressive growth of premalignant lesions separates the interior of the tumor from the surrounding blood supply, which results in hypoxic conditions with low partial pressures of oxygen [29]. Around the tumor, a metabolic shift called the “Warburg effect” appears. There is an increase in glycolytic metabolism involving the conversion from glucose to lactate compared to oxidative phosphorylation preferred by normal cells. The increased production of lactate results in extracellular acidity (pH 6.5–6.9), which promotes ECM degradation and cell invasion [30,31]. Nonmalignant endothelial cells, fibroblasts, macrophages, and T cells have also been observed in the stroma surrounding solid tumors along with metabolic interactions between stromal cells and cancer cells [32,33].

Based on the understanding of the characteristics of the tumor microenvironment, “smart” nanocarriers have been developed in order to overcome pathophysiological barriers. They are expected to offer high accuracy, selectivity, and high sensitivity for the targeted delivery of loaded drugs in a predictable or controlled manner. Aspects such as the penetration of cytotoxic drug-loaded nanocarriers into solid tumors, intracellular drug delivery, and reaching a concentration within the therapeutic window at the target site also aid in reversing multidrug resistance [34,35]. Targeted drug delivery may allow drug activity to be localized specifically at the target tumor site rather than in other organs in order to improve treatment outcomes and reduce systemic side effects. Antibodies, vitamins, peptides, or sugars that recognize tumor-specific antigens, receptors, or transporters have been developed for the active targeting of conjugated drugs [36]. Materials responsive to stimuli such as pH, light, temperature, magnetic field, redox activity, or enzymes at the target tumor site are needed for the design of smart nanocarriers [37,38,39,40]. For instance, pH-responsive chitosan (CS) and bioresorbable polydioxanone containing polydopamine nanospheres can be used for such applications [41,42]. Drugs released from the pH-sensitive nanocarriers do not accumulate at pH 7.4 but accumulate in an acidic tumor environment [43,44,45,46]. Nanomaterials responsive to photothermal stimuli generate heat to destroy tumor cells in response to near-infrared (NIR) light irradiation [47,48,49,50]. Drug delivery nanoplatforms that can perform drug release in response to a redox reaction or specific enzyme have also been developed [51,52,53,54,55,56,57,58,59,60,61,62] (Table 2).

**Scheme 1:**
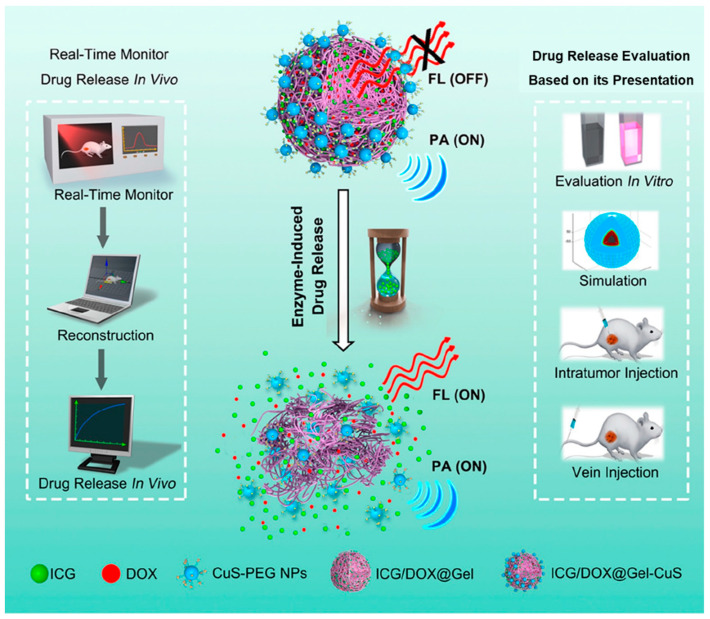
Development of enzyme-activatable indocyanine green (ICG)/doxorubicin (DOX)@Gel-CuS nanomedicines (NMs) with a core-satellite architecture made of gelatin nanoparticles (NPs) loaded with near-infrared fluorescent ICG and chemo-drug DOX and coated with “satellite” CuS NPs. The DOX release from ICG/DOX@Gel-CuS NMs can be monitored by fluorescence (FL) and photoacoustic (PA) dual-modal imaging in a real-time manner due to core-satellite architecture and degradability of the gelatin matrix in response to proteases overexpressed in tumor. The FL of ICG, which was initially quenched within intact NMs, increases according to the DOX released from NMs (reproduced with permission from [64], Copyright 2019 American Chemical Society).

The anticancer DDS that can react with enzymes and control the release of DOX was developed by using cystamine-modified gelatin [63]. Oil-in-water microemulsion containing cystamine-modified gelatin, Fe_3_O_4_, and DOX were prepared for delivery by utilizing the EPR effect at the tumor site. Cystamine-modified gelatin released DOX, which was in response to glutathione (GSH) and matrix metalloproteinase (MMP). Since the amount of GSH and MMP is overexpressed in cancer tissues compared to that in normal tissues, the developed microemulsion can control the release of DOX in cancer tissues. Controlled drug releases induced by GSH and MMP improved tumor therapy and drug monitoring using T2 magnetic resonance imaging (MRI) were possible due to the properties of Fe_3_O_4_. While the drug release was regulated by enzymes, monitoring the drug concentration was important for determining the efficiency and prognosis of cancer treatment. Core-satellite nanomedicines for accurate real-time monitoring of enzyme-induced drug release were also developed [64]. This nanomedicine was developed using DOX and indocyanine green (ICG), which acted as medical diagnostics and were loaded with surrounding CuS nanoparticles (ICG/DOX@Gel-CuS nanomedicines). CuS nanoparticles (NPs) generate a photoacoustic signal independent of the physical status of the ICG/DOX@Gel-CuS nanomedicines (NMs). This independent property aids in the real-time tracking of nanomedicines. In contrast, ICG fluorescence depends on the physical status of ICG/DOX@Gel-CuS NMs. The stable state of ICG/DOX@Gel-CuS NMs results in quenching and interference with ICG fluorescence. ICG/DOX@Gel-CuS nanomedicines and the controlled release of DOX were monitored up to 24 h. The application of ICG/DOX@Gel-CuS NMs resulted in significantly reduced tumor sizes without toxicity (Scheme 1).

## 3. Organic Nanocarriers for Anticancer Therapy

Depending on the constituent materials, organic nanocarriers for anticancer therapy are broadly classified as polymer-based nanoformulations, lipid-based nanoformulations, and protein-based or virus-based nanoparticles.

### 3.1. Polymer-Based Nanocarriers

#### 3.1.1. Polymeric Nanoparticles

Synthetic and natural polymers have been applied for controlling the fluidity of liquids, film coating in pharmaceutical formulations, controlled drug release, targeted drug delivery, improvement of the bioavailability (BA) of drugs, and biomedical implants [65]. Compared to liposomes, polymeric nanoparticles demonstrate the advantage of controlling the stability and release of loaded drugs [66]. Poly(lactide-co-glycolide) and poly(cyanoacrylate) are used for the preparation of drug delivery nanocarriers. In the polymerization reaction involving a dispersion system, nano-sized particles can be produced by using surfactants above the critical micelle concentration.

**Figure 2:**
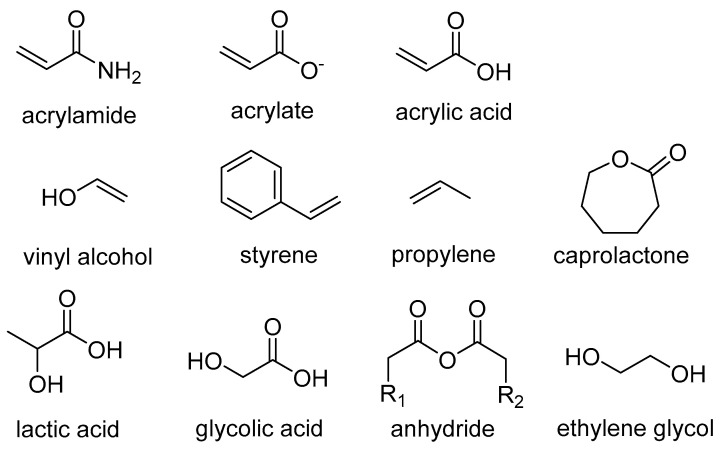
Basic unit of polymers used for polymeric nanoparticles.

Polymeric nanoparticles are nanometer-sized particles composed of polymers that are exploited as carriers of therapeutic agents for invasive and non-invasive routes of delivery [67]. Such systems are designed to respond to a variety of internal stimuli around the tumor tissue, such as pH, reductive environment, temperature, and enzyme concentration, in order to maximize drug delivery at the target site [9]. These nanoparticles undergo physiochemical structural changes when exposed to these stimuli, thereby losing their well-defined nanoarchitecture and releasing drugs directly into tumor cells [10]. Polymers used to prepare such formulations consist of at least one moiety derived from acrylamide, acrylate, and acrylic acid and other moieties derived from cellulose derivatives, poly(vinyl alcohol), polystyrene, polypropylene, polycaprolactone, poly(lactic acid-co-glycolic acid) (PLGA), polyanhydride, etc. [11]. Grafting of hydrophilic polyethylene glycol (PEG) to polymeric strands allows modification with the targeting ligand or self-assembly with the pH-responsive polymer [68]. Coating nanoparticles with the proper PEG derivative is a strategy for decreasing the interaction of nanoparticles with plasma opsonins and uptake by mononuclear phagocytes [69,70] (Figure 2).

#### 3.1.2. Micelles

Surfactants in the aqueous solution aggregate in response to certain concentration and temperature conditions to form micelles. Micelles within the particle diameter range in which drug-loaded micelles are expected to accumulate in the tumor tissue via the EPR effect can be selected for cancer therapy. The size and physical properties of micelles can be controlled by adjusting molecular weight, chemical composition, and component ratio.

**Figure 3:**
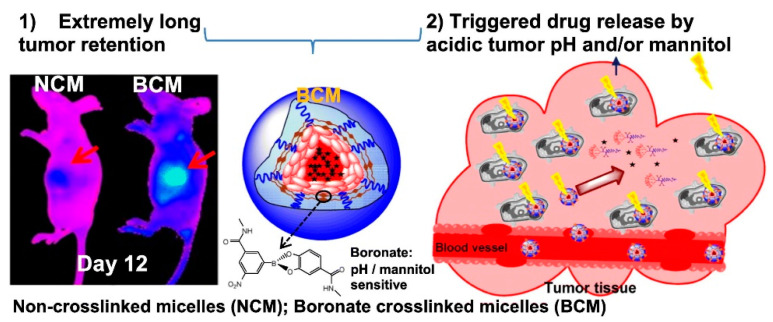
This is schematic illustration of long tumor retention and dual-sensitive boronate cross-linked micelles (BCM) for cancer therapy (reproduced with permission from [74], Copyright 2017 Elsevier). Boronate ester bonds formed between boronic acids and cis-diol catechols (such as mannitol) are responsive to pH and/or mannitol. Near-infrared fluorescence imaging (excitation = 625 nm, emission = 700 nm) was used for visualization of drugs in vivo.

Polymeric micelles can be modified for stimuli responsiveness and active targeting for cancer drug delivery. Depending on the manufacturing method, they can be used as a carrier of hydrophilic or hydrophobic drugs, specifically in chemotherapy. GeneXol-PM^®^ was approved by the FDA in 2007 as a polyethylene glycol (PEG)-PLA-based polymeric micelle, which is a carrier of PTX and is effective in treating breast cancer and lung cancer [71,72]. Anti-cancer effects or physicochemical properties can be improved via the modification of micelles or conjugation. Cavalcante et al. developed pH-sensitive micelles in order to enhance antitumor activity and reduce toxicity [73]. DSPE-PEG_2000_, oleic acid, and DOX were used to manufacture micelles. Oleic acid forms an ion pair with the DOX amine group at pH 7.4. As oleic acid becomes protonated at pH 5 or lower pH, the ion pair is unpaired, and the micelle becomes unstable, resulting in the release of DOX. Upon examining the release profile of DOX for 24 h, approximately 30% and 60% of DOX were released at pH 7 and pH 5, respectively. When the tumor size was compared to that of the control group, the micelles comprising the ion-pair of DOX with oleic acid were significantly smaller. In the case of micelles without the ion-pair of DOX and oleic acid, an anticancer effect was observed; however, the difference was not significant when free DOX was used. These observations suggest that the ion pair of DOX and oleic acid releases DOX in response to pH. Xiao et al. developed boronate cross-linked micelles (BCM) that demonstrated an extremely long tumor retention period. BCM showed much better stability than the non-crosslinked micelles [74]. Furthermore, BCM showed excellent stability under in vitro conditions in the plasma at physiological pH but lost their structure under acidic pH and the addition of mannitol (Figure 3). BCMs were retained in the tumors for 12 days after drug administration, and the size of the tumor and tumor-associated mortality were statistically reduced.

#### 3.1.3. Dendrimers

Dendrimers exhibit a regular branched structure comprising symmetric small molecules around the core and polymeric branch units [75]. Depending on the structure of branching units, which demonstrate a tree shape or star shape, polymeric dendrimers show three-dimensional spherical or rugby ball shapes. Divergent synthesis and convergent synthesis are conventional preparation methods for dendrimers [76,77]. The Diels–Alder reaction, thiol–ene reaction, or alkyne–azide reaction have been also attempted for the design and synthesis of dendrimers [78,79,80].

Polymeric dendrimers made from polyamidoamine are expected to enhance the water solubility of hydrophobic drugs, which are enclosed in the internal space or bound to a functional group on the surface of the dendrimers [81]. The surface functional groups of dendrimers can also be chemically modified for targeted drug delivery and to decrease toxicity [82]. Dendrimers can also be used as gene carriers. Na et al. developed a dendrimer-type bio-reducible polymer as a therapeutic gene delivery carrier [83]. Small interfering RNA (siRNA) is an RNA molecule that interferes with the expression of a specific gene and is primarily examined for applications in cancer treatment [84]. Na et al. used cystamine bisacrylamide diaminohexane (ABP), which is an arginine-grafted bio-reducible polyamidoamine (PAMAM), to synthesize a dendrimer-type bio-reducible polymer (PAM-ABP). An anti-vascular endothelial growth factor (VEGF) siRNA was loaded into PAM-ABP. VEGF, which has been studied for cancer treatment, is a mitogen that plays an important role in the improvement of angiogenesis [85]. Angiogenesis and lymphangiogenesis have been observed during the growth of cancer, which are attributed to the overexpression of VEGF. These developments are primarily responsible for progression and metastasis. The anti-VEGF antibody prevents cancer growth and metastasis by blocking angiogenesis and lymphangiogenesis. The size of anti-VEGF-loaded PAM-ABP was 116 nm, and VEGF gene silencing was more effective than PEI/siRNA in HT1080, A549, and Huh-1 cell lines with no cytotoxicity of PAM-ABP observed under in vitro conditions. Otis et al. developed antibody-conjugated dendrimers in order to target cancer cells [86]. Otis et al. used an anti-human epidermal growth factor receptor-2 (HER-2) antibody (Herceptin) to functionalize the dendrimer with targeting properties. Her-2 was bound to the dendrimer surface by using many functional groups, and a target diagnostic material was developed by placing gold nanoparticles (AuNPs) and gadolinium (Gd) inside the dendrimer. The encapsulation of AuNPs and Gd was confirmed by UV-vis and ICP-OES. The dendrimer showed a high absorption rate in the A549 cell line, which was Her-2 positive, and the authors suggested that a clinical approach was possible with respect to incorporating the effects of herceptin and CT/MRI dual modus imaging.

### 3.2. Lipid-Based Nanoformulations

#### 3.2.1. Liposomes

Lipid-based nanoformulations have been used as nanocarriers for chemotherapeutics as well as vaccine adjuvant-delivery systems [87]. In addition to cancer, there are various indications for them, including hepatitis, influenza, and COVID-19 [88].

Liposomes, which are biodegradable vesicles with lipid bilayers composed mainly of phospholipids, have been clinically used as delivery vehicles for drugs such as DOX and daunorubicin [89]. Lipophilic drugs can be trapped in the liposomal lipid membrane, and hydrophilic drugs can be dissolved in the inner liquid core of the liposome. Liposomes have also shown potential for the delivery of genes, proteins, and peptides [90,91].

Methods such as thin-film hydration, sonication, extrusion, and microfluidic mixing are used for manufacturing liposomes [92,93,94]. After dissolving lipids and drugs in an organic solvent, a thin film is prepared using a rotary evaporator, and liposomes are prepared via rehydration. Thereafter, uniform liposomes can be manufactured by additionally performing ultrasonic treatment or extrusion of liposomes.

In order to lower the uptake of liposomes by reticuloendothelial system (RES) cells and to prolong the circulation half-life of loaded drugs, PEG was incorporated into the liposomal membrane [95]. The liposomal membrane can be modified with targeting moieties or antibodies specific to the target cell [96]. We can also design stimuli-responsive liposomes for improving drug delivery and anticancer efficacy [62]. Liposomes can be administered via both oral and intravenous routes. Kim et al. reported a three-fold increase in the BA of docetaxel (DTX) [97]. The liposome coated with Eudragit^®^ significantly inhibited the release of DTX at pH 1.2 of the gastric environment. In contrast, docetaxel was quickly released at pH 6.8 without any statistically significant difference when compared to the release from non-coated liposomes. The oral BA of free docetaxel was 1.91 ± 0.232% (Table 3). Interestingly, the oral BA of liposomes coated with Eudragit^®^ was 5.92 ± 1.31, which is higher than that of the control group. This suggests that the Eudragit^®^-coated liposomes are expected to stably deliver drugs, including DTX, and enhance oral absorption.

#### 3.2.2. Solid Lipid Nanoparticles

Solid lipid nanoparticles (SLNs), which are spherical nanoparticles composed of lipids that possess a solid lipid core matrix, have also been investigated for cancer treatment with active nanomedicine development in order to overcome the limitations of conventional anticancer drugs [98]. SLNs have been well established because they enhance the efficiency of drug delivery and can deliver hydrophobic and hydrophilic drugs without toxicity issues [99,100]. Smith et al. studied a novel DDS with SLNs in order to improve stability and avoid the rapid metabolism of 5-Fluorouracil (5-FU) [101]. SLNs loaded with 5-FU were prepared by high-pressure homogenization. Glycerol monostearate, mPEG2000-DSPE, compritol, or precirol was used as the matrix, and Tween 80, lecithin, and poloxamer were used to stabilize SLN. SLNs loaded with 5-FU were cytotoxic to HCT-116 cells, with less than 10% cell viability observed at the concentration of 25 μM. The 5-FU release profile of SLN was analyzed. Most of the free 5-FUs were rapidly released within 4 h. However, in the case of SLN, 5-FU was slowly released over 8 h, with only 70% of the drug released over 24 h. In addition, Western blotting was used to confirm the inhibition of factors closely related to cancer growth, such as epidermal growth factor receptor and protein kinase B (AKT), in HCT-116 cells. NOD/SCID mice were subcutaneously inoculated with HCT-116 cells (5.7 × 106 cells) in order to develop a colorectal cancer xenograft model. The mice received an intraperitoneal (i.p.) injection of SLN every alternate day for two weeks. Pharmacokinetic profile analysis showed that the area under the curve from the starting point to *t_max_* (AUC(0-t)) of 5-FU SLNs was 5-fold higher (54.7 ± 3.2) than that of the free 5-FU (15.5 ± 1.9). The tumor size was evaluated for 30 days. The tumor size of the group that was administered SLN was significantly smaller (approximately 200 mm^3^) than that of the untreated group (approximately 350 mm^3^).

### 3.3. Virus-Based Nanoparticles

Virus-based nanoparticles (VNPs) are being examined in many studies and are a great candidates for the development of smart therapeutics because of their virus-specific advantages such as cell penetration, strong gene expression, possible opportunities for engineering, and modification of functional groups [102,103]. Among virus-based particles, adeno-associated virus (AAV)-based particles, which are used for continuous gene expression and the induction of mild or very low immune responses, have been well characterized [104]. AAV/VEGF-Trap was studied for the prevention of tumors and pulmonary metastasis by blocking angiogenesis, and effective inhibition of tumors with a single intravenous administration was demonstrated by Lu et al. [105]. RNA interference is a promising strategy for cancer treatment. It can interfere with the growth of cancer cells, improve apoptosis of tumor cells, and prevent cancer metastasis. The study was conducted with a breast cancer therapeutic agent that introduced short hairpin RNAs into AAV [106]. AV-based carriers (rAAV-PSMA2sh) showed the highest shRNA knockdown efficiency in HEK293T cells, and the tumor size was also significantly smaller in animal experiments conducted twice a week for four weeks. Virus-based nanoparticles were applicable for the treatment of various diseases [107]. Owing to the unique characteristics of the virus, it is primarily used as a smart system to deliver genes [108]. Hence, the number of clinical trials examining the efficacy of such methods increased rapidly, and technologies involving virus-based nanoparticles are being further advanced.

## 4. Inorganic Nanocarriers and Hybrid Nanoplatforms for Anticancer Therapy

In this century, advances in synthetic chemistry have provided tools for synthesizing a wide range of inorganic nanoparticles. Owing to their inherent properties, we can exploit their traits and use them as drug delivery agents as well as imaging representatives. Their robust infrastructure allows us to encapsulate more than one functional component with different capabilities, which enhances the potency of the system. Owing to advantages such as non-toxicity, biocompatibility, hydrophilicity, and high stability over organic materials, they are widely used in the field of drug delivery [109].

Common inorganic nanoparticles can be classified into three distinct categories:

- Inorganic nanoparticles derived from metals, such as gold, silver, iridium, and platinum, which show phenomenal resistance towards oxidation;
- Magnetic nanoparticles (MNPs) are mainly derived from 3d and 4f metals, such as Fe_3_O_4_ and Gd_2_O_3_;
- Fluorescent nanoparticles such as silicon-based quantum dots and lanthanide-based upconversion nanoparticles [110,111].

Although significant efforts have been made to use them as imaging agents and for enhanced radiotherapy and other biomedical applications in oncological systems, their usage as drug delivery vehicles has garnered little interest. Low toxicity, high cellular uptake, and nonimmunogenic response have contributed to their use in the field of DDS. For particles with sizes within the nano region, especially those based on inorganic materials, distinct properties have been observed compared to their bulk counterparts. In the field of biological sciences, the amount of magnetism exerted by nanocomposites, as well as their sizes, shapes, and other optical properties, can substantially alter the expected outcome [112].

The immunoferritin method, mercury-based method, techniques involving uranium, and immunocolloidal methods involving gold have been reported earlier for antibody markers. Since then, the market for the biomedical industry has grown and is vastly dictated by bio-conjugated compounds incorporating inorganic metals [113,114,115,116]. In recent times, we have seen significant advances in the field of DDSs, as progress in nanotechnology has enabled us to develop various nanoparticles without any size restrictions. Carbon nanotubes, fullerenes, and graphene quantum dots (GQDs) also play an important role [117,118,119]. We can attach a drug to the surface moiety of graphene, which will be used for targeted drug delivery and the reduction in side effects and toxicity as well as cost-effectiveness. Owing to their specific chemical characteristics, inorganic nanoparticles are stable and not subject to microbial attack; therefore, novel pharmaceutical therapies are now being developed based on a nanoparticle platform.

The synthesis of inorganic nanoparticles involves many techniques, including hydrothermal methods, microwave-assisted methods, laser ablation, template synthesis, spark discharge, and sputtering. This is mainly applicable to dry particles and nanoparticles disseminated in the liquid phase. Nano-sizes in materials can also be achieved by reducing the size from micro to nano at the atomic level [120].

Owing to their inherent properties, iron oxide nanoparticles (INP) are highly used when it comes to DDSs associated with MNPs. Giorgia Pastorin et al. have reported studies showing the release of sorafenib from INP coated with PVA-LDH and PEG-LDH and synthesized by using ammonia as a base. X-ray spectroscopy, Fourier-transform infrared (FT-IR) spectroscopy, transmission electron microscopy, scanning electron microscopy (SEM), dynamic light scattering, high-performance liquid chromatography, UV-visible spectrophotometry, thermogravimetric analysis, and vibrating sample magnetometry have been used for characterization. In the cytotoxicity assay performed against normal 3T3 fibroblast cells, no toxicity was observed after 72 h with different concentrations of nanoparticles. While mediating anticancer activities against HepG2 cells in acidic media, the nanoparticles showed significant cytotoxicity, making sorafenib-loaded and surface-modified INPs potent candidates for magnetic DDS [121].

In 2019, Rostamizadeh et al. demonstrated the efficiency of MNPs coated with PEGylated curcumin in cancer therapeutics [122]. They exploited the magnetic targeting characteristics of the nanoparticles. First, they synthesized dicarboxylated PEG using a standard method in which acetone was used to dissolve PEG, and the entire reaction was completed under ice-cold conditions. The next step involved the synthesis of PEGylated curcumin based on NHS/EDC chemistry followed by the synthesis of MNPs coated with PEGylated curcumin. A coprecipitation method was used for MNP. This system can be used as a pH-dependent magnetic drug carrier.

### Superparamagnetic Iron Oxide Nanoparticles (SPIONs)

Another study reported the use of modified superparamagnetic iron oxide nanoparticles (SPIONPs) using block copolymers such as PCL-PEG-PCL. The study involved the preparation of nanoparticles by using the same principle; ammonium hydroxide was used as a base. In the case of co-polymer synthesis, the authors used the ring-opening polymerization technique, where Sn(Oct)_2_ was used as a catalyst. In this case, 5FU was used as the drug. The double emulsion method (water/oil/water) was used for encapsulation inside the nanoparticle. The encapsulation efficiency was measured by using a spectrophotometer at 266 nm. The study reported drug encapsulation efficiency of up to 90%. Based on the characterization studies, FT-IR showed an exact peak at 580 cm^−1^ for distinct Fe-O bond stretching in Fe_3_O_4_, while XRD data showed an inverse spinel structure. SEM analysis corroborated the well-aggregated structure of the nanoparticles. In the in vitro drug release studies, SPIONPs released a maximum amount of 5-FU (33.1%), and the overall release was 84.1% in 48 h depending on the acidic pH (pH 5.4) [123].

In DOX-encapsulated Fe_3_O_4_ nanoparticles, hexaethyl cellulose (HEC), NCC, and polyvinylpyrrolidone could be used for surface modification. In the cell viability assay performed against hFOB cells, all surface-modified nanoparticles showed reduced toxicity compared to free Fe_3_O_4_ nanoparticles. Among them, the lowest toxicity was shown by particles coated with HEC, although cationic charges are more prone to be cytotoxic. The drug loading efficiency was 71% for HEC-coated nanoparticles and 89% for PVP-coated nanoparticles. Examination of cytotoxicity was performed with the MCF-7 cell line. Equal toxicity was observed for both DOX-loaded nanoparticles and pure DOX at higher concentrations in the range of 400–200 µg/mL. PVP-coated nanoparticles with doses <250 µg/mL showed no noteworthy toxicity towards normal cells (*p* > 0.05); however, HEC-coated nanoparticles showed higher toxicity towards cancer cells, making them a perfect candidate for drug delivery carriers [124].

Another DOX carrier was reported in 2019. Here, Fe_3_O_4_@SiO_2_ nanoparticles were used for which their surfaces were modified by (NIPAM-co-GMA) (PNG) chains via surface-initiated reversible addition-fragmentation chain transfer (SI-RAFT) polymerization. Hydrazine was used to modify the polymer bushes. Encapsulation efficiency peaked at 78%. At high concentrations of DOX-and DOX-conjugated nanoparticles, high cytotoxicity was observed against the cancer cell line (HeLa cells). The study was performed with stimuli-responsive nanocarriers showing enhanced biocompatibility [125].

Surface-modified superparamagnetic iron oxide nanoparticles (SPIONs) with nonporous SiO_2_ (@SiO_2_), mesoporous SiO_2_ (m@SiO_2_), and a mixture of both have been reported for targeting human lung cancer cells A549 and BEAS-2B. They used tetraethyl orthosilicate as a precursor and hexadecyltrimethylammonium bromide as a pore former for m@SiO_2_ only. While treating cancer cells with SPIONs, we have to be cautious about one side effect: Dissolution of SPIONs and its possible release of iron at the target site can encourage tumor growth. These coatings significantly altered the surface morphology of SPIONs, and during experimentation, it was evident that a single m@SiO_2_ coating constrained iron release from the nanoparticles by more than 10-fold when compared to that observed with unmodified SPIONs. Based on the AAS-ET measurements, it was evident that both nanoparticles were effectively assimilated by these two cells. Both nanoparticles strongly affected the proliferation of BEAS-2B cells; however, in both cases, no cytotoxicity was observed. Therefore, it was concluded that both nanoparticles were cytocompatible with lung epithelial cells [126].

Lipoamino acid-coated SPIONs have also been reported. Studies performed with human hepatocarcinoma cell line Hep-G2 showed that compared to the naked SPIONs, surface-modified cells showed less cytotoxicity and higher cell viability (more than 100%). Cell viability increased in a time-dependent manner. Lipoamino acid-coated nanoparticles showed growth-enhancing effects due to the supervised release of ionic iron into cells, rendering them highly biocompatible and a good targeted delivery system for cancer therapeutics [127].

CS-coated iron oxide MNPs with phytic acid (PTA) were reported in 2017 by Dena Dorniani et al. to develop nanocarriers, which showed impressive anticancer activity against human colon cancer cells and no toxicity towards healthy fibroblast cells. However, the loading of PTA was significantly lower (12.9%), which was confirmed via spectroscopic studies. The PTA release profile suggested that the release of PTA was significantly lower than that of the physical mixture of PTA sodium salt of CS and MNP, corroborating the fact that the aforementioned nanocomposite can be used as a controlled-release drug delivery carrier. The release, which follows pseudo-first-order kinetics, reached 93% within 56 h when the pH was 4.8 and 86% within 127 h when the pH was 7.4. These effects were attributed to the instability of the compound in acidic media, which suggests a bias towards the acidic pH environment. With respect to pure compound (IC_50_ = 188.5 µg/mL), the nanocomposite shows much higher cytotoxicity against HT29 cells as well as lower IC50 value (IC_50_ = 45.63 µg/mL). Hence, this nanocarrier is both selective and more efficient than pure PTA [128].

The cytotoxic, antiproliferative, and apoptotic effects of carboxylated quercetin-conjugated SPIONs with surface modification with (3-aminopropyl) triethoxysilane (APTES), folic acid (FA), and carboxylated PEG have been demonstrated against FA receptor-negative A549 and FA receptor-overexpressed MCF-7 and HeLa cells, which have paved the way for the development of candidate nano drugs against breast and cervical cancer cell lines [129]. Heat shock protein inhibitor-loaded silica-coated Fe_3_O_4_ nanoparticles with carboxyl functional group modification showed thermotherapeutic and chemotherapeutic effects against lung cancer stem cells [130]. Temperature-dependent PTX release from magnetic solid lipid nanoparticles based on iron oxide was reported, which showed increased BA of the drug as well as controlled release under magnetic hyperthermia conditions [131]. Transferrin-modified PLGA nanoparticles loaded with PTX were reported for MCF-7 breast cancer and U-87 glioma cells in vitro, showing the highest cytotoxicity with respect to free nanoparticles and PTX [132].

By utilizing the magnetoelectric properties of nanoparticles, their use as nanocarriers is also a smart approach in modern biopharmaceuticals that exploit magnetic and electric field interactions during drug loading and delivery to a specific target site [133,134]. The PTX-based drug carrier prepared using CoFe_2_O_4_ and BaTiO_3_ with surface functionalization by glycerol-monooleate demonstrates a non-zero magnetic moment, which helps to move the carrier in the circulatory system owing to the external magnetic field. In vitro and in vivo studies demonstrate that upon treatment with an intravenous injection of the PTX-loaded drug carrier followed by the application of an external magnetic field for three weeks, nude mice with xenografted ovarian carcinomas were completely cured. This claim was supported by IR imaging histology studies performed via energy-dispersive spectroscopy and immunohistochemistry [135].

## 5. Electrospinning for Production of Nanofibers in Bulk

**Scheme 2:**
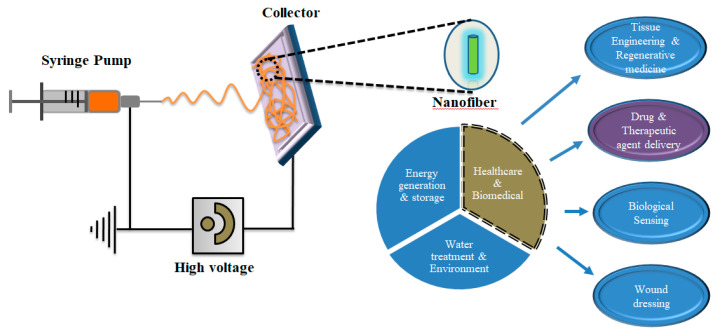
Schematic illustration of a traditional electrospinning process and various healthcare and biomedical applications of nanofibers (reproduced from [142], MDPI 2019).

In order to understand the interaction between nanomaterials and biological systems in terms of biocompatibility and minimal biological toxicity as well as to achieve desired therapeutic efficacy, researchers have been investigating a diverse range of nanomaterials, such as zero-dimensional (carbon dots, quantum dots, and GQDs), one-dimensional (nanowires, nanorods, nanotubes, and nanofibers), and two-dimensional (graphene oxide, transition metal oxide, MXenes, etc.) nanomaterials along with polymer-based nanoparticles for diagnosis, imaging, and therapy [136,137]. Among them, nanofibers have been sought as promising delivery vehicles because of their tunable porosity and easy functionalization with biological molecules. These remarkable characteristics make them a robust and ideal candidate for water and environmental treatment, energy generation and storage, and biomedical applications [138,139,140,141,142] (Scheme 2).

Electrospinning is a versatile technique that is easy to perform on a bulk scale with a diverse range of sizes from nanometer to micrometer scale with a variety of materials such as inorganic, hybrid (organic–inorganic), and natural and synthetic polymers [143]. This method is typically selected because of controllable particle diameter, minimum consumption of solution, easy handling, and cost-effectiveness for large-scale production. The physical characteristics of the electrospun nanofibers, such as fibrous structure, surface morphologies (hollow, dense, or porous), fiber diameter, and surface-to-volume ratio, can be monitored by using the following parameters: (i) processing parameters (electric potential, feeding rate, and flow rate); (ii) solution properties (molecular weight of polymer, viscosity of solution, dielectric constant, conductivity, and surface tension); and controlled post-processing parameters (heating rate and heating temperatures) [144,145]. Electrospinning techniques can be categorized into five methods: (i) coaxial electrospinning, (ii) blend electrospinning, (iii) melt electrospinning, (iv) emulsion electrospinning, and (v) gas jet electrospinning. Nanofibers prepared by blend electrospinning can be used for bursting release, while sustained release can be achieved by using coaxial and emulsion methods with a core-shell structure. Nanofiber fabrication by melt electrospinning produces highly ordered particles with relatively larger diameters [146].

Nanofiber-based delivery systems have demonstrated to be potent candidates among other delivery systems because of their superior loading capacity and high surface area for the desired functionalization. In the last few decades, FDA-approved PTX has become a vital and effective drug for cancer treatment. The delivery of PTX to accurate tumor sites with higher loading efficiency for cancer treatment remains the most challenging issue because of its poor solubility. In order to overcome this problem, Xu and co-workers developed nanofibers fabricated using PTX and succinic acid, which showed the highest loading efficiency of ~89% with a controlled release profile. Moreover, these nanofibers significantly inhibited the proliferation of human lung adenocarcinoma cells in both in vitro and in vivo evaluations [147]. Another study showed that PTX-loaded surface-modified mesoporous hollow stannic oxide nanofibers (SFNFPs) enhanced anticancer activity in liver cancer treatment. SFNFP significantly improved the dissolution of PTX in the release experiment performed under in vitro conditions. The dissolution rate of SFNFP was 8.34-fold higher compared to that of free PTX within 5 min. The cumulative release rate of free PTX was approximately 16.77 ± 2.00% within 1 h while it was 80.00 ± 2.64% for SFNFP. In the in vivo study, SFNFP suppressed tumor growth significantly, and the inhibition rate was 67.00 ± 0.40%, which could be an ideal delivery vehicle for liver cancer treatment [148].

Norouzi et al. demonstrated salinomycin-loaded electrospun nanofibers for the first time during local glioblastoma therapy and tumor recurrence prevention. The nanofibers were composed of biodegradable poly lactic-co-glycolic acid polymers developed via electrospinning. These fibers were stable for approximately 30 days and showed a sustained release of approximately 80% of salinomycin in 4 days, and the remaining drug was released within two weeks of the initiation of nanofiber degradation. The sali-loaded nanofibers were capable of generating intracellular reactive oxygen species and upregulating the expression of Rbl1 and Rbl2 tumor suppressor genes as well as caspase 3, which can induce cancer cell apoptosis. These nanofibers demonstrated significant cytotoxicity against U251 glioblastoma cells compared to free Sali, which could be a potential candidate for brain tumor degradation [149].

## 6. Conclusions and Future Perspectives

With continued innovation in medicinal chemistry, the development of nanomaterials for biomedical applications has shown promise for applications in biological systems, and nano DDSs have shown great potential in cancer research. Even though anticancer drugs such as DOX, PTX, and curcumin are used for treatments, their performances are limited when it comes to the targeted drug delivery to the tumor site. Stimuli-sensitive nanocarriers for controlled drug release have garnered interest in cancer therapy. Bispecific antibody-conjugated nanotherapeutics can be used to enhance targeting efficiency. Recently, many approaches have been developed along with the design of inorganic nanoparticle-mediated DDSs. Although a significant amount of research on smart nanocarriers has been performed over time, improvements are needed for surface fabrication as well as enhanced efficacy in order to push them further into clinical trials. Further research is required for the design of smart nanosystems with stability and specificity. Lipid nanocarriers for RNA delivery have also been developed. They can protect naked RNA and deliver RNA into cells [150]. Smart immune-nanomedicines can play a role in cancer therapy as well as COVID-19 management with minimized off-target side effects [151].

Electrospun nanofibers are attractive and promising candidates for cancer therapy owing to the manufacture of nanofibers from the laboratory at the industrial level. Owing to their remarkable properties, nanofibers have been used extensively in biomedical applications. Although production and applications are promising, further development and improvements are required for enhancing the efficacy of therapeutic systems.

## Figures and Tables

**Table 1:** Pharmaceutical aspects of cancer nanomedicines approved by USA or Europe.

Name (API)	Approved Indication	Formulation and Administration Route	References
Doxil/Caelyx (doxorubicin)	Ovarian cancer, multiple myeloma	PEGylated liposome and intravenous infusion	[13]
DaunoXome (daunorubicin)	Kaposi’s sarcoma	Liposome and intravenous infusion	[11]
Ontak (Engineered fusion protein combining diphtheria toxin with interleukin-2)	Cutaneous T-cell lymphoma	Proteinaceous nanoparticle and intravenous infusion	[18]
Myocet (doxorubicin)	Metastatic breast cancer	Liposome and intravenous infusion	[12]
Eligard (Leuprolide acetate)	Advanced prostate cancer	Polymeric nanoparticle and subcutaneous injection	[19]
Abraxane (paclitaxel)	Non-small cell lung cancer, metastatic breast cancer, metastatic pancreatic cancer	Albumin-bound nanoparticle and intravenous infusion	[14]
Marqibo (vincristine)	Acute lymphoblastic leukemia	Liposome and intravenous infusion	[15]
MEPACT (mifamurtide)	Osteosarcoma	Liposome and intravenous infusion	[17]
Onivyde/MM-398 (irinotecan)	Metastatic pancreatic cancer	PEGylated liposome and intravenous infusion	[16]
VYXEOS/CPX-351 (cytarabine and daunorubicin)	Acute myeloid leukemia	Liposome and intravenous infusion	[10]
NBTXR3/Hensify (radiotherapy)	Squamous cell carcinoma	Hafnium oxide nanoparticle and intratumoral injection	[8]
NanoTherm (Iron oxide)	Brain tumor	Magnetic nanoparticle and intratumoral injection	[9]

API: active pharmaceutical ingredient; PEG: polyethylene glycol.

**Table 2:** Recent publications on stimuli-responsive drug delivery nanocarriers for smart anticancer therapy.

Stimulus	Formulation	References
pH	Polymersome by self-assembling of a carboxyl-terminated polyethylene glycol amphiphile	[40]
pH	Lectin-conjugated mesoporous silica nanoparticle	[43]
pH	Phosphorylcholine polymer micelle	[44]
pH	Polymeric micelle based on heparin-α-tocopherol conjugate	[45]
pH	Self-assembling polypeptide and calcium phosphate	[46]
Photothermal	Dipalmitoyl phosphatidylcholine liposome	[47]
Photothermal	Copper sulfide nanoparticle	[48]
Photothermal	Silica-coated silver-gold nanoshell	[49]
Redox	Zwitterionic cross-linked micelle based on a penta-block copolymer	[38]
Redox	Inorganic nanoparticle functionalized by organic group, polysaccharide, or peptide	[51]
Redox	Liposome with disulfide-phospholipid conjugate	[52,53,62]
Redox	Polymeric nanomicelle	[55,56,57,58]
Enzyme	Micelle formed from two amphiphilic block copolymers	[39]
Enzyme	Monostearin/amorphous calcium carbonate nanoparticle	[59]
Enzyme	Self-assembled protein nanoparticle	[60]
pH, redox, and enzyme	Gelatin-encapsulated magnetic nanoparticle	[63]

**Table 3:** Pharmacokinetic parameters after oral administration of free docetaxel (DTX) (20 mg·kg^−1^) and Eudragit^®^-coated liposomal DTX (10 mg·kg^−1^) (reproduced with permission from [97], Copyright 2018 Springer Nature).

Parameter	DTX (20 mg·kg^−1^, Mean ± SE)	Eudragit-Coated Liposomal DTX (10 mg·kg^−1^, Mean ± SE)
*t_max_* (min)	110 ± 10.0	90.0 ± 9.49
*C_max_* (μg·mL^−1^)	0.0112 ± 0.00193	0.00981 ± 0.00169
*K_a_* (min^−1^)	0.0609 ± 0.0257	0.0349 ± 0.0165
*K* (min^−1^)	0.00168 ± 0.000726	0.000995 ± 0.000181
*t_1/2_* (min)	567 ± 181	818 ± 182
AUC (μg·min·mL^−1^)	6.98 ± 0.846	10.8 ± 2.39
*V_β_* (mL·kg^−1^)	44,745 ± 14,275	64,605 ± 14,381
BA (%)	1.91 ± 0.232	5.92 ± 1.31

DTX: docetaxel; SE: standard error; *t_max_*: time of maximum drug concentration; *C_max_*: maximum drug concentration in plasma or serum; *K_a_*: absorption rate constant; *K*: elimination rate constant; *t_1/2_*: elimination half-life; AUC: area under the curve; *V_β_*: volume of distribution in the β-phase; BA: bioavailability.

## Data Availability

Not applicable.
